# Swedish stakeholders’ attitudes towards challenges and opportunities regarding support efforts to older persons’ conditions for living with a high quality of life in ordinary housing: a panel study

**DOI:** 10.1186/s12877-026-07719-w

**Published:** 2026-05-30

**Authors:** Mirkka Söderman, Carl Johansson, Lena-Karin Gustafsson

**Affiliations:** Division of Caring Science, School of Health, Care and Social Welfare, Mälardalen University, Box 325, Eskilstuna, 63105 Sweden

**Keywords:** Older people, Municipal health and social care, Ordinary housing, Person-centered care, Sweden

## Abstract

**Background:**

Increasing amounts of older people are able to stay in their own homes, with the opportunity and desire to continue their lives that way, though they often have diverse age-related needs. This means healthcare staff has to have right conditions to work with advanced holistic person-centered care.

**The aim:**

Was to identify panel consensus regarding attitudes toward challenges and opportunities in healthcare support for older people living at home.

**Methods:**

Data was collected through three rounds of chronological mail rounds, with 45 statements presented to an panel (*n*=20) of that represents the network and decision makers surrounding care and support for older people in ordinary housing on municipal level.

**Results:**

All statements achieved a certain level of consensus. Today's care recipients are both older and frailer with major healthcare needs, and family members are in turn in need of recovery. Housing is also not always adapted to the increased needs of older people. Also, there is a lack of communication and collaboration between stakeholders’ design of nursing efforts, access to human resources, and use of health and welfare technology.

**Conclusions:**

It is important to consider both individual and organizational conditions when planning an extended home care for older people. This should take place in team cooperation to also ensure that personnel resources are used for the right tasks, and that digital solutions are optimized in line with the wishes and expressed needs of the older people and their families.

## Background

Healthcare for older people in terms of the legal regulation and efforts are similar in the Nordic countries but differ from other European countries in some ways [1] (in this context, the term “efforts” denotes formal care and support interventions, corresponding to the Swedish concept “insatser”). For example, the availability of service delivery in Sweden is dependent on the municipalities’ specific organizations and conditions. Though all of them include home service, home health care, special accommodation for the older people, senior housing, or other support from the municipality, an increasing number of older people are able to stay in their own homes, with the opportunity and a desire to continue their lives that way. Including a desire for maintained independence and the ability to influence one’s everyday life [2]. This growing phenomenon needs to be addressed throughout the Western world and therefore needs massive illumination from many different angles. Authorities highlight a structural shift where advanced medical care is increasingly provided in the home, placing high demands on municipalities to ensure competence and patient safety. Older people often have a diversity of health-related needs, which means that a key factor is to strengthen and enable healthcare professionals to work with an conscious and evident person-centered approach to care. To achieve a caring practice in line with person-centred care a framework of Prerequisites (staff attributes) and care environment (context) are necessary conditions to enable a Person-centred process (the care activities) which ultimately facilitate or hinder a care practice with respect for the person and his/her self-determination and understanding (PCP) [3, 4].

Similarly, older people living in ordinary housing wish for person-centered care, to be seen as a person and not a number, and to obtain improved self-confidence and independence [5, 6]. Caring relationships with professionals are significant [5–10] but the specific meaning of the relation is a unique, subjective experience [11], and a prerequisite for feeling involved in the care and experiencing security for the families. Older people and their families appreciate continuity with few healthcare professionals involved [7, 8], and good communication to be able to participate in decision-making [10]. Older people desire more information to understand and manage their medical condition, to be strengthened in managing their situation and gain greater control over their lives [5]. Medical products and technical aids are also mentioned as a way to deal with physical limitations [7] as well as e-Learning [5]. Older people often have a positive attitude to health and welfare technology if it promotes their ability to stay at home [12], and if they live far from their relatives, “smart homes” give an increased sense of security [13]. Family members, e.g., spouses, often wish to share the responsibility of care but do not always know how to do it [8]. They need to receive information about the range of services and support about housing, daily activities, and disease-specific services, as well as how they can take part in this [6, 8, 10].

While previous research has focused on isolated aspects of home care (e.g., technology acceptance or caregiver burden), there is a lack of consensus on how these factors interact to create a sustainable care environment. This study contributes by synthesizing knowledge across organizational boundaries, from decision-makers to providers, to identify the holistic conditions necessary for person-centered care in ordinary housing. Therefore, the aim of this study was to identify panel consensus regarding attitudes toward challenges and opportunities in healthcare support for older people living at home. The study addresses the following research questions: (1) What represents the critical structural conditions (challenges) for older people to remain in ordinary housing with a high quality of life? And (2) What working methods and interventions do panelists identify as necessary (opportunities) to ensure person-centered support in the home?

## Methods

The study was carried out during spring 2023 among panelists, i.e., a panel of network and decision makers surrounding care and support for older people in ordinary housing on municipal level who participated in the study. This study was inspired by the Delphi method description of Hasson, Keeney and McKenna [14] and Kenney, Hasson and McKenna [15]. Participants were given the opportunity to identify consensus around what can be considered as challenges and opportunities regarding support efforts in favor of older people living in ordinary housing since the consequence of healthcare actions constitute a significant – positive or negative – contribution to older persons’ conditions for living with a high quality of life in their own homes. Also, to reason for alternatives to meet older people’s different expectations of the conditions for remaining in ordinary housing and suggest ways of working to support this in the home.

### Sample

The identification and thus selection of participants was performed with a targeted selection strategy to find participants who would truly constitute support and decision-makers in the field of support from society regarding health, care and social welfare. A central contact person at the Municipal Health Care Administration of a medium-sized municipality in Sweden suggested persons to contact. Inclusion criterion was that the participants would represent organizations at the local level in the municipality such as various decision makers, aid workers, support functions, group leaders and others with responsibility for various forms of healthcare efforts for the older people living at home.

Forty-two panelists were identified and invited to the study. Of these, 18 women and two men consented to participate and answered the survey in the role of specialist nurse, nurse specialized in healthcare development and improvement, occupational therapist, unit manager, operations manager, aid manager, case manager, relative consultant, or active within the Volunteer Center, see Table 1.

**Table 1:** Demographic characteristics of the participants

#	Role	Gender
1	Specialist nurse	Female
2	Unit manager	Female
3	Not specified	Female
4	Case manager	Female
5	Case manager	Female
6	Case manager	Female
7	Occupational therapist	Female
8	Unit manager	Female
9	Occupational therapist	Female
10	Operations manager	Female
11	Operations manager	Female
12	Unit manager	Female
13	Specialist nurse	Female
14	Operations manager	Male
15	Family consult	Female
16	Aid manager	Female
17	Occupational therapist	Female
18	Aid manager	Female
19	Active within the Volunteer Center	Male
20	Not specified	Female

### Data collection and analysis

The data collection was carried out through three rounds of electronic questionnaires, so-called chronological mail rounds. Statements in each subsequent round were based on the answers from the previous round, see Table 2. According to the Delphi methodology of panel statement analysis, the procedure was repeated until consensus occurred among the panelists. The process of developing consensus took place through three email rounds via a questionnaire with free text responses which were then thematized into several statements. A selection of statements with higher consensus was chosen to strengthen consensus in the final round. The panelists received a link to a web-based survey created using the `Survey & Report´ tool.

**Table 2:** In brief, the process of developing consensus

Mailround	Panelist	Type of questions and information in the questionnaire	Analysis	Statements
1	20	7 Open responses	Thematic	45
2	20	45 Statements, Likert-scale	Descriptive analysis	20
3	20	20 Statements, Likert-scale	Descriptive analysis	-

The procedure in the survey was transparent for the participants, i.e., the answers from each round were shared, albeit anonymously. In this way, the panelists reviewed each other’s answers, but their identity was hidden to reduce the effect of influence based on each other’s perceptions and opinions that would arise in, for example, a group interview [16]. Letting the panelists take part in each other’s anonymous answers is a method of obtaining an answer to research questions that are not influenced by personal power relations or hierarchies that could otherwise constitute bias in, for example, group interviews. 

### Mail round 1: open survey questions

The survey in the first email round contained both background questions and four overall questions, with an introductory question: “What challenges exist for everything to work well for older persons and their family to stay at home?”, with room for comprehensive answers and the opportunity for further comments with three closing questions. The web-link to the questionnaire was sent via the central contact person at the Health Care Administration and was open for three weeks at the end of February-March 2023.

The responses to the open-ended questions from the first round were analyzed via thematic analysis developed by Brown and Clark [17, 18] and turned into statements. The analysis method provides a systematic procedure for generating codes and themes when analyzing the qualitative data set. Codes are then the smallest units of analysis that capture, in relation to the purpose, potentially interesting findings in the data. Through the codes, themes are built up which are the core of the analytical observations. Through thematic analysis, you summarize the content and identify the most important themes in relation to the purpose/research question. The analysis followed recommended steps for thematic analysis:

1. The answers to the open questions were read several times to familiarize the researchers with the material.
2. In step two, an initial code generation was made, after which themes emerged from the generated codes.

An inductive approach was chosen to capture specific priorities of the panel. Following review, defining, and naming (see Braun & Clarke [17, 18]) seven themes emerged: Caring interventions, Human resources, Health and welfare technology, Housing, Communication/collaboration, Care recipient, Family. The themes were operationalized into statements (see Table 3) closely aligned with data; e.g., the data extract “support efforts are not individualized enough” was coded as person centered care, themed as lack of person-centered care, and operationalized into statement “Support efforts are not person-centered enough”. The statements that were used in round 2 varied in scope and perspective (client, professional, manager) which reflects the heterogeneity of the panel. No pilot testing was conducted, but the formulations remained as close as possible to the original quotes to ensure authenticity. This process was used to ensure the quantitative phase is grounded in the panelist’s insights.

**Table 3:** Statements (*n* = 45) formulated after thematization of the open answers and adapted to a five-point likert scale for the panel to rate

#	Statements formulated according to round 1	Consensus (mean) round 2	Selected statements	Consensus (percent) round 3 Sometimes	Consensus (percent) round 3 Often	Consensus (percent) round 3 Always
	Caring interventions		-			
1	Support efforts are not person-centred enough	2,9	-			
2	The individual has not been fully involved in the planning of their efforts*	3,0	To round 3	33,3	50,0	-
3	Care recipients and their families have different views on care and care planning	2,8	-			
4	The alarm group/alarm situation does not work optimally	2,1	-			
5	The distribution of resources varies for different groups and overrides the right to equal treatment	2,4	-			
6	Difficult to adjust times for home care according to the care recipient’s needs	3,1	To round 3	44,4	50,0	-
	Human Resources		-			
7	Continuity among the staff may be lacking in home care	3,5	-			
8	Several persons/staff are involved in each care recipient’s efforts	3,6	To round 3	11,1	83,3	5,6
9	Staff competence may be lacking in home care	3,4	To round 3	38,9	61,1	-
10	Language difficulties among staff make communication difficult	3,3	To round 3	22,2	83,3	-
11	Staff shortages and recruitment difficulties are a problem in home care	3,5	To round 3	16,7	77,8	22,2
12	The care recipient’s home can fail as the staff’s work environment	2,6	-			
13	There is a need for increased resources in the form of meeting places for care recipients	2,9	-			
	Health and Welfare Technologies					
14	It is too slow with the development of health and welfare technology within municipal health and care	3.3	To round 3	42,1	36,8	10,5
15	It is too long and complicated processes to introduce technical solutions that already exist	3,5	-			
16	Difficult to bring in digital solutions as compensation for care recipients who have already received other support	3,2	To round 3	33,3	66,7	-
17	Sometimes aid assessors do not inform about which welfare technology solutions are available	2,9	-			
18	There is an insecurity even among home care staff to introduce digital solutions to the care recipient	2,6	-			
19	The care recipients are not so used to technology that they can search for themselves and thus make informed decisions	3,3	To round 3	5,6	83,3	11,1
	Housing					
20	The nature of the dwelling, the hygiene areas are not adapted to the care recipient’s needs	3,2	To round 3	33,3	72,2	
21	The home’s kitchen is not adapted to the care recipient’s needs	2,7	-			
22	Many of the aids received are not adapted to the care recipient’s specific home	2,4	-			
23	Necessary aids do not fit in the home**	2,7	-			
24	The property the care recipient lives on does not have an elevator	2,5	-			
25	Housing options are lacking in the open housing market	2,6	-			
	Communication/Collaboration					
26	There is a lack of knowledge and coordination about who should inform about what	3,1	-			
27	There are boundaries between region and municipality that create barriers	3,1	To round 3	47,4	52,6	
28	There are gaps in communication between healthcare providers	3,4	To round 3	57,9	42,1	
29	There is a lack of overall support in the possibility of receiving healthcare at home	2,8	-			
30	The homecoming chain is not working properly***	2,9	-			
31	The region’s actors do not keep what they promised when at homecoming	3,0	-			
	Care recipients					
32	The target group today is older and thus more fragile	3,5	To round 3	21,1	73,7	5,3
33	There is a high risk of falling that requires close access to staff	2,9	-			
34	Increased physical illnesses require large nursing needs and health care efforts	3,5	To round 3	31,6	68,4	-
35	It is very difficult for persons with cognitive impairment to manage themselves at home	3,6	To round 3	21,1	78,9	-
36	There are great risks involved in letting persons with cognitive impairment stay at home	3,5	-			
37	Severe illness entails that the care recipient does not feel safe despite extensive home care efforts	3,1	-			
38	Today, there is widespread isolation among older care recipients	3,5	-			
39	Many experience loneliness and that home care is the only social contact	3,4	To round 3	31,6	68,4	-
	Family					
40	More relief options are needed so family members can recover	3,2	To round 3	35,0	35,0	20,0
41	Family members have also a right to their home, which is disregarded when planning interventions	3,0	To round 3	68,4	15,8	10,5
42	There are gaps in knowledge about how relatives get help and support in being able to live at home longer	3,0	-			
43	There is an insecurity among relatives about where to turn	3,3	To round 3	52,6	47,4	-
44	There is an inaccessibility for families to receive emergency support	2,9	-			
45	Lack of family members/relatives is an obstacle to being able to stay at home	3,1	To round 3	47,4	26,3	5,3

Lickert Scale: Never, Seldom, A few times, Often, Always. Consensus in mean value is shown for the positioning of statements after round 2, and percentage for the selected statements (*n* = 20) after round 3. Response option Never is not presented for round 3 as no one in the panel had agreed to that option

*In this context refer to formal care and support interventions, ** In Swedish it means that there is no room, *** Refers to the process from discharge from hospital to the person returning home

### Mail rounds 2 and 3: evaluation of statements

The second questionnaire included 45 statements constructed from a thematization based on the open answers and adapted to a five-point Likert scale for the panelists to rate (Never, Seldom, Sometimes, Often, Always) [19], see Table 3. The questionnaire was sent via the central contact person at the Health Care Administration and was open for three weeks in April 2023. Of the 20 people who chose to answer the first survey, all answered both the second and third survey. Round 2 responses were analyzed using descriptive statistics (percentage, mean, standard deviation). From round 2, statements from each theme were selected based on the mean value ≥ 3.0 of the positioning to round 3. To reduce redundancy and ensure a focused questionnaire for the final round, statements that reached consensus in round 2 but were conceptually overlapping were collapsed (item 7, 15, 31, & 36). This consolidation explains why some high-scoring items did not proceed independently into round 3.

The third and final survey included 20 statements selected based on a higher agreement among the participants when it came to statements in the second survey. This third survey was also sent via the central contact person at the Health Care Administration and was open for three weeks during May 2023. In this way, the research subjects could take part in each other’s anonymous survey responses and then change or further develop their reasoning about claims. The responses from round 3 were analyzed with descriptive statistics (percentage, mean, standard deviation) and are presented in percentage. None of the 20 statements from round 3 reached unequivocal consensus, ≥ 75% agreement [20], as shown in Table 3. However, all 20 statements from round 3 reached some consensus at least *sometimes* this was the case presented in the statement.

### Ethics

The project was approved by a Regional Ethical Review Board in Sweden (Dnr 2022-02629-01). The project was conducted following the Declaration of Helsinki [21] and informed consent was obtained from all participants following the guidelines for GDPR [22]. Furthermore, the invited participants were told that participation was voluntary and that the collected data would be handled to ensure that only authorized persons have access to it. They were also told they were free to withdraw from the specific study without having to explain. A submitted response was considered to consent to participate.

## Results

The statements reported above, and where the panelists found consensus, are presented in the running text below. The results are based on the analysis of both qualitative and quantitative data, i.e., statements based on free-text responses, and percentages and mean values of positioning of statements. The statements were thematized into seven key themes to describe the older people’s conditions for remaining in residence as well as working methods which were judged to be decisive for support in ordinary housing. Themes such as Human Resources, Care Recipient, Family, and Housing primarily relate to structural conditions, while Caring intervention, HWT, and Communication/collaboration relate to working methods. By having a broader perspective on the results and bringing together alternative responses to statements, a consensus was found about all selected statements for round 3, i.e., these aspects occur to some degree.

### Caring interventions (6 statements)

It appeared from the panelists’ statements that support efforts for the older people are not considered to be person-centered enough, e.g., the older persons have not been fully involved in the planning of their efforts or are not familiar with the arrangement: “The individual does not know when the home care staff will arrive, the individual has not been allowed to participate in planning how” (P17). It appears that there often seems to be a lack of knowledge on the part of the older person/family about how to get help at home and what kind of help is available so that the older person can live longer in ordinary housing. The older people and families may also have different points of view when it comes to care planning.

Furthermore, homecare was not perceived by the panelists to function satisfactorily for older people or their families. This was illustrated by the fact that it is difficult to adapt times for homecare to the needs of older people. The alarm system intended for unexpected events was not considered to function optimally as it seems to take too long from alarm to help, i.e., to the healthcare staff’s responsiveness to the need for support. The older person might raise the alarm between the planned visits for various reasons, and it leads to a feeling of insecurity if the older person needs help with, e.g., a toilet visits within a reasonable time. A view was expressed that the healthcare staff need to be available and have time to listen to and see the needs of the older person.

Another aspect is that when units specialized for certain diagnostic groups are built, ordinary homecare is perceived as depleted.

*"We are building up several units that are supposed to be specialized, but instead we are depleting the foundation of homecare …We are then building our own little culverts that do not benefit most of the older persons"* (P4).

This means that the distribution of resources varies for distinct groups of older people with specific needs. That overrides the right to equal treatment that exists under legislation when healthcare is not designed in a way that suits the individual needs of all older home-dwelling people.

### Human resources (7 statements)

The panelists considered there is both a staff shortage and difficulties in recruiting new healthcare staff. This leads to a lack of continuity among the healthcare staff, and there might be several different people visiting older people’s homes. It also appears that older persons according to the panelists, have stated that it is stressful with all the temporary healthcare staff, and it creates insecurity: “Many of the older persons say that it is so difficult with all the temporary workers who come to them, some feel insecure as there are often new temporary workers” (P5).

Furthermore, the panelists believed that healthcare today places great demands on the staff for healthcare to function. Therefore, there is a need to increase the skills among healthcare staff. That is especially the case with advanced home health care. Communication between the older persons and the healthcare staff is also hampered by staff language deficiencies due to multicultural workforce: “Lack of communication between care recipients/families and the healthcare staff. They don’t get to know each other.” (P).

When it comes to the working environment of healthcare staff, the panelists explained that the older persons’ homes can create a poor working environment, becoming too difficult for the healthcare staff, despite the decisions of the aid officer.

### Health and Welfare Technology (HWT) (6 statements)

It is highlighted by the panelists that the older people are not sufficiently tech-savvy to search for themselves and thus make informed decisions about HWT. During aid assessments, the older persons do not always receive information about the technological solutions available today (communication and vision aids, and aids used for movement and activities of daily living). Once older people have received support in another form, it is believed that it is difficult to introduce digital solutions as compensation, for something currently performed by family members.

It also appears that the development of HWT services within municipal healthcare is perceived by the panelists as slow, often because there are too long and complicated processes in introducing technical solutions, even if those are already available: “Technology that takes over what I need help with, AI (artificial intelligence) doesn’t exist today, the technological leap hasn’t come yet” (P4). When older people must wait for HWT solutions, they often get those at such a late stage that they don’t have enough time to learn how to use them and feel comfortable with such solutions.

### Housing (6 statements)

The panelists stated that the older person’s ordinary housing is not always or cannot be adapted to their needs. For example, when it comes to stairs that are not adapted or lack of a lift, cramped apartments and toilets and shower rooms that are not adapted to the needs of the older people. Furthermore, the panelists believed that aid received does not always fit or is not adapted to the older person’s homes. For example, toilets can be difficult to enter with assistive devices and impossible to support the older persons in: “Sometimes, a person might be able to live at home longer and avoid moving into a nursing home if their accommodation had been more suitably adapted” (P10). In addition, it is highlighted that there is a lack of housing alternatives such as more variants of secure housing, both rental and condominium options.

### Communication/collaboration (6 statements)

The panelists stated that there is a lack of overall information as support for opportunities to receive healthcare in ordinary housing, especially when returning home after hospitalization periods but also regarding healthcare efforts from primary care. Partly since there are restrictions drawn between regional and municipal healthcare that create barriers but also since there is a lack of communication between care providers. Sometimes, there is a lack of knowledge and coordination about who should inform about what. Furthermore, it is perceived that the homecoming chain does not work, e.g., that the regional actors do not keep what they promised at homecoming or do not send what is needed for the first 24 h at home such as such as medicines or wound dressings: “If the patient does not receive the right aids or interventions from the hospitals in the first 24 hours, the patient often just turns the door and has to go back to the hospital”(P6).

### Care recipient (8 statements)

The panelists emphasize that the target group in ordinary housing today is characterized by extensive medical complexity. The older persons may have illnesses that affect the ability to both provide healthcare and to be able to manage living in ordinary housing: “Illnesses that affect everything from cognitive impairment as well as physical illnesses that require major nursing needs and healthcare efforts” (P8). A critical aspect raised is the high risk of safety incidents such as falls or situations where persons with dementia leave their homes unaccompanied. This complexity creates a structural challenge where the patient’s safety is compromised due to the gap between their high needs and the level of monitoring available in ordinary housing. This also creates concern among their families.

It is also emphasized by the panelists that today there is widespread social isolation among the older persons: “Loneliness impairs the joy of life which can impair physical and mental abilities and create greater nursing needs” (P20). They emphasized that many of the older people included experience loneliness which creates an increased need for social meeting places. The home service might be the only human contact for many older people, and they are there only for short periods for specific interventions, e.g. basic care measures. And on the other hand, it creates anxiety for both the older people and their families when several different staff visit the older people. Some older people do not allow healthcare staff to enter their home, which makes the situation in ordinary housing untenable.

### Family (6 statements)

The panellists perceived that lack of family members or relatives was stated as the biggest challenge to being able to remain in ordinary housing. It appeared from the statements that there are gaps in knowledge about how and what kind of help families can receive in ordinary housing to get support for the older person with healthcare needs to be able to remain in ordinary housing: “Many caregivers who struggle without knowing that there is an opportunity for relief. Family caregivers are a support that we must take care of” (P9). In some cases, families may need support more urgently, but there is a lack of availability. Nevertheless, more relief options are needed so that the families can recover: “That we would have the opportunity to enter at an earlier stage to relieve the burden on relatives to last longer at home and so that situations do not escalate as far as becoming unsustainable” (P20). The family members do also have a right to their own homes, which can be overridden when planning healthcare interventions for the home-dwelling older people. It can give a feeling of limited privacy that healthcare professionals come to their homes, sometimes several times a day.

## Discussion

The aim of this study was to identify panel consensus regarding attitudes toward challenges and opportunities in healthcare support for older people living at home. In response to the first research question regarding structural conditions, the consensus indicates that high staff turnover and fragmented organization constitute critical barriers to continuity and safety. Addressing the second research question concerning working methods, the result highlights that active pedagogical interventions and improved communication strategies are necessary to bridge the gap between the older persons capabilities and the demands of the home care environment. The panelists described seven areas that can function better or worse to make a prolonged living arrangement in older people’s ordinary housing more available. While previous research has argued for the importance of older people’s participation in decision-making [10], our results show that the decision-makers are worried about older people not being fully involved in planning interventions. Part of the low involvement is described as facilitated by communication barriers, and part of it is due to low levels of knowledge of what interventions are available. The Swedish Board of Health and Welfare [23] has described that when welfare interventions are unavailable or caregivers are considered of poor quality, often due to high turnover and low continuity among care personnel and poor care relations between care personnel and the care recipient, the closest family is incentivized to step in involuntarily. Such situations, in turn, are associated with increased stress and a caring relationship that is not primarily conditioned by love but by necessity [24]. Adding to this complex process, the results also show that the panelists describe relatives as being uninformed about what support their municipality and regional healthcare organizations can offer. These findings inform our first research question regarding structural conditions. Viewed from the lens of a Person-cantered process-framework (PCP) [y], the identified deficiencies in housing and organization indicate a compromised Care environment. A structural instability that prevents the formation of effective staff relationships and continuity, which are essential contextual factors for safe care. Therefore, decision makers must prioritize strategies that stabilize the care environment. I.e. securing staff continuity and adapting housing solutions to create the necessary prerequisites for ageing well at home.

One intervention the panelists describe as beneficial for family caregivers is using welfare technology that allows a presence in the older person’s life while at a distance. Such arrangements have been described as offering a sense of security [13]. Older people have requested more knowledge about these products to increase their participation in implementing welfare technology [6, 8, 10]. The panelists in this study added that the level of tech-savviness also influences the participation of older people, as more knowledge means a better ability to make informed decisions in the vetting process.

Further, it is perceived as challenging to introduce new HWT solutions if other non-technical solutions cover a need. The results are ambiguous in the sense that the panelists describe a situation where older people are described as hard to motivate to accept welfare technology as a part of care, while at the same time, the technology is described as a factor that enables the closest family to participate in care and enhance a feeling of safety. The results seem to point towards a need for a pedagogical approach to HWT that can both give the sense of security that is described by Turjamaa [13] and that is described by the panelists in this study while also giving enough details around its implementation that a person can make an informed decision around its implementation. Making informed decisions around implementing care interventions is important for participation in the care process, which might be self-evident but also is necessary for person-centered care [4]. From this perspective, the pedagogical gap becomes an issue that needs to be solved, not only for the trustfulness of the technology but to live up to the values of person-centered care [3]. This informs our second research question regarding working methods. To ensure person-centered support, working methods must shift from passive information giving to a pedagogic dialog. Within the theoretical framework [3] this represents a shift in the Person-centered process, specifically focusing on shared decision making and engagement. Our results suggest that without active pedagogical interventions to bridge the gap between older persons capabilities and the demands of the home, these person-centred processes cannot be fully realized.

Another critical aspect of the structural conditions (research question1) is the transition between hospital stay and home. When a need for care interventions arises, a person has sometimes previously received hospital care until ready to be discharged. At this point (in the Swedish healthcare system), the responsibility of care is transferred from the region to the municipality of the older person [25]. This transformation has been described by various Swedish researchers and authorities as complex as both stakeholders need to be ready to hand over and take over the care responsibility simultaneously [26, 27]. The results of this study enhance this problem as the panelists describe a situation where they receive a patient that is ready to be discharged by the region but not ready to be received by the municipality. Here, a lack of communication makes it hard to prepare to receive the patient and begin care in the home. Adding to this dilemma is the fact that older people’s homes are sometimes hard to adapt to the various needs of carers. If the responsibility for care is transferred prematurely, there is a risk that the adaptations in the older person’s home are not yet ready. Finally, the panelists also describe that communication between the stakeholders is unsatisfactory, further adding to the difficulties in preparing the home for care.

Regarding the structural conditions (RQ1), the panelists also emphasize the critical role of family. The care recipient and his or her family are important factors for homecare. The panelists raise concerns about older people’s safety. They describe issues around fall prevention and compromised security when people with severe dementia can leave the house alone. The panelists also raise concerns about an older person’s potential social isolation and describe that the professional care staff may be the only human contact the older person has in a day. And at the same time, they have also described concern about having too many professionals working with a person living at home; some are even there specifically to break up social isolation. It is unclear what kind of isolation the panelists refer to when they agree on the statement [38 *Today*,* there is widespread isolation among older care recipients*]. Previous research suggests that social isolation among older persons in Sweden is complex. Öberg et al. [28] problematized that in the public debate, few older people are voiced over their social situation. Instead, studies have shown that experienced loneliness among older people does not increase with age from younger middle age and onward [29–32], with the exception for the absolute oldest people. This argument supports the panelist’s statement that older people do have many contacts if the meetings with care personnel are seen as breakers of social isolation. However, on the other hand, Swedish research has shown that older adults are more exposed to existential loneliness as existential needs are often neglected by professionals and rarely documented as a need by healthcare professionals [33]. Our point is that it is hard to know what the panel means when they describe social isolation as a problem for older people in municipal care, and the phenomenon deserves more research, not necessarily the experience of loneliness itself, but what care professionals make of it.

Finally, the panelists describe the relationship between municipal healthcare professionals, the older person and the older person’s family as important for the older person’s ability to have care interventions in the home. The family is described as important support, a claim that has been acknowledged by Swedish authorities to be of great importance for care in general but also care in the person’s home [23]. The authorities have recently published a strategy to strengthen the support of family carers. However, the panelists in this study report concerns over the lack of family members, an issue that has also been described by Johansson et al. [34]. Johansson et al. suggest that older adults commonly report having family-integrated care as an important goal for them to feel safe and happy, but those who lack that resource could compensate with other kinds of social capital, for example, friends or organizations that they are part of. If family members are present and live with the older person, the panelists report a dilemma where family members also have the right to their home when interventions are planned.

### Strengths and limitations

The invitation to participate in the study was sent to 42 persons, and as only 20 chose to participate, that needs to be considered when interpreting the results. Also, only two of the 20 panelists were men. This was certainly the case because healthcare sector is a female-dominated work field and most of the panelists worked in connection to that field. The same gender distribution is also assumed to exist among people who did not consent to the study. On the other hand, the spread in terms of organizational level was wide, from specialist assistant nurse to operational manager, and to different professions. Among the panelists, there were also a few people with an indirect connection to the field, such as those active within the Volunteer Center and case managers at the municipality. This spread among participants can be seen to balance the low response rate and gender distribution.

Although some guidelines suggest a minimum of 30 participants for robust statistical analysis, Delphi literature widely supports panel sizes between 15 and 30 when the focus is on specialized expertise rather than statistical representativeness [35, 36]. However, the sample size of 20 in this study constitutes a limitation for the quantitative analysis. In a panel of this size, each respondent accounts for 5% of the total variance, making descriptive statistics such as means and percentage thresholds sensitive to individual outliers. Consequently, the quantitative results should be interpreted as an indicator of consensus within this specific panel, rather than statistically generalized results.

The panelists seem to have considered the topic of the study important, and it engaged them given that the response rate was 100% between the three response rounds. A few of the thematized statements about the older people’s conditions for remaining in ordinary housing and working methods to support that achieved consensus according to the usual lowest level, i.e., 75% which is not a usual limit within the Delphi methodology, but on the other hand, the results showed that all the thematized aspects occur to some degree.

This study was conducted in a smaller Swedish city, which means that it may not be transferable to all contexts. However, this study is an important contribution as it raises the current issue of challenges and opportunities for the aging population who wish to remain in their homes.

## Conclusions

This study enriches the already existing body of knowledge and confirms and gives validity to some of the knowledge that is needed in our work with older persons’ conditions in ordinary housing, even though it does not reveal totally new knowledge. It is important to consider both individual and organizational conditions when planning an extended stay at home for t this growing group of older people in need of support at home. Today’s home care recipients are both older and weaker with major healthcare needs, and the family members are in turn in need of recovery. The communication and collaboration between different decision-making levels regarding the design of healthcare interventions need to be improved. This should take place in team cooperation to also ensure that personnel resources are used for the right tasks, even across organizational boundaries, in line with the wishes and expressed needs of the older people and families. The housing situation needs to be adapted to the needs of the older person. Furthermore, working methods regarding digital solutions must shift from passive information giving to a pedagogic dialog, ensuring the older person can make informed decisions about their own care environment.

## Data Availability

The datasets generated and/or analyzed during the current study are not publicly available due to Swedish regulations and laws but are available from the corresponding author on reasonable request.

## Abbreviations

HWT: Health and Welfare technology
